# Chiral carbon dots: synthesis, optical properties, and emerging applications

**DOI:** 10.1038/s41377-022-00764-1

**Published:** 2022-03-27

**Authors:** Aaron Döring, Elena Ushakova, Andrey L. Rogach

**Affiliations:** 1 Department of Materials Science and Engineering, and Centre for Functional Photonics (CFP), City University of Hong Kong, 83 Tat Chee Avenue, Kowloon, Hong Kong SAR China; 2 Center of Information Optical Technologies, ITMO University, Saint Petersburg, 197101 Russia; 3 Shenzhen Research Institute, City University of Hong Kong, 518057 Shenzhen, China

**Keywords:** Nanoparticles, Nanophotonics and plasmonics

## Abstract

Carbon dots are luminescent carbonaceous nanoparticles that can be endowed with chiral properties, making them particularly interesting for biomedical applications due to their low cytotoxicity and facile synthesis. In recent years, synthetic efforts leading to chiral carbon dots with other attractive optical properties such as two-photon absorption and circularly polarized light emission have flourished. We start this review by introducing examples of molecular chirality and its origins and providing a summary of chiroptical spectroscopy used for its characterization. Then approaches used to induce chirality in nanomaterials are reviewed. In the main part of this review we focus on chiral carbon dots, introducing their fabrication techniques such as bottom-up and top-down chemical syntheses, their morphology, and optical/chiroptical properties. We then consider emerging applications of chiral carbon dots in sensing, bioimaging, and catalysis, and conclude this review with a summary and future challenges.

## Abstract (web summary)

This review focuses on chiral carbon dots, introducing their chemical syntheses, morphology and optical/chiroptical properties, and considering their emerging applications in sensing, bioimaging and catalysis.

## Graphical abstract

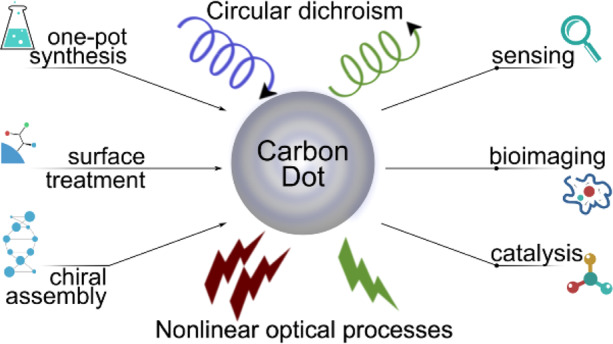

## Introduction

The terms *chiral* and *chirality* originally stem from geometry and were defined by Lord Kelvin as “*I call any geometrical figure, or group of points*, ***chiral****, and say that it has* ***chirality*** *if its image in a plane mirror, ideally realized, cannot be brought to coincide with itself*.”^1^ In this terminology, chirality refers purely to the object’s geometry and does not necessarily infer any information on the physical and chemical properties of a chiral material. In chemistry, chiral molecules are defined as left- or right-handed enantiomers forming sets of stereoisomers. Many naturally occurring molecules, with amino acids being prominent examples, are chiral. Importantly, building blocks of life^2^, such as proteins, nucleic acids, glycans, and lipids are dominated by an asymmetrically distributed number of enantiomers. RNA has been reported to spontaneously replicate in a homochiral system, but this replication can be blocked by the presence of the opposing enantiomers. This is one of the current challenges in biophysics, which raised questions on the emergence of RNA^3^. Furthermore, plenty of bulk inorganic materials may exhibit random chirality due to asymmetry in the distribution of their defects^4^.

With the emergence of nanotechnology, novel pathways opened up towards the fabrication of artificial chiral objects, such as nanoparticles and their assemblies. We refer the interested readers to several reviews devoted to the chirality in colloidal semiconductor quantum dots^5–7^, metal nanoparticles^8^, nanoparticle assemblies^9,10^, liquid crystals^11^, and metamaterials and metasurfaces^12^. A great interest in chiral artificial systems is attributed to a plethora of potential future applications, which range from the optical activity control of detectors/emitters^13–15^, to enantioselective catalysis^16^, sensing^17,18^, bio-applications^6,19^, and medical treatment of diseases^20,21^. The latter area is of particular interest, since nanomaterials can provide both diagnostic and therapeutic functions combined with controllable physicochemical properties.

In the last decade, luminescent carbon nanoparticles have appeared as a novel class of carbon-based nanomaterials. As the research on these nanoparticles intensified, categorization and terminology used for these materials became a point of debate^22^. Designations such as graphene quantum dots, carbon dots, carbon nanodots, and carbon polymer dots have been used in the literature depending on the synthetic method and structure of resulting nanoparticles, albeit sometimes interchangeably^23^. In this review, we selected the term *carbon dots* to denote them in general terms, unless specified otherwise.

Carbon dots have been studied extensively in recent years, in particular due to their light-emissive characteristics, and the fact that they are based on less toxic material (carbon) as compared to traditional semiconductor quantum dots which often include heavy metals such as cadmium or lead^24^. Carbon nanomaterials in general are considered to be environmentally friendly, and their precursors are common and rather abundant, so they can be produced at a low cost^25^. Fluorescent carbon dots have already found applications in light-emitting devices^26,27^, light harvesting^28^, bioimaging^29,30^, as well as in catalysis^31^ such as in hydrogen production^32^. Moreover, carbon dots have a high thermo- and photostability, and their emission can be very efficient, with reported quantum yields of up to 90%^33,34^. In analogy with an evolution from spherical semiconductor quantum dots towards 1D quantum rods and 2D nanoplatelets^35,36^, fluorescent colloidal carbon nanoparticles of other shapes have recently been reported, such as carbon nanorods^37^, rolls, and belts^38^. At the same time, both the energy structure and anticipated emission mechanisms of carbon dots are distinctly different from those of the classical semiconductor quantum dots. Quantum confinement of energy levels and the related optical transitions, which are the common characteristics of classical quantum dots^39^, is only one of the possible scenarios which have been mostly reported for graphene quantum dots. Indeed, the origin of light emission in carbon dots is much more complex, and is related to the co-existence of multiple emissive centers within carbon dots, which is also reflected in their rather broad, excitation-dependent emission^23,40^. Four major emission origins of carbon dots have been highlighted in literature: (i) fluorescence from constituents of carbon dots which are similar to fluorescent organic molecules^41,42^; (ii) core-state fluorescence from conjugated π-domains which can be related to quantum confinement of those^43,44^; (iii) surface-state related emission originating from interactions between the carbon core and surface atoms^45–47^; and (iv) crosslink enhanced emission reported for polymer dots^22,33^. We notice that the latter mechanism has only recently come into focus of scientific investigations; it has been reported that the fluorescence of organic molecular fragments within carbon dots (which could even be non-fluorescent in other conditions) could be greatly enhanced through suppression of nonradiative relaxation (vibrational and rotational modes) via their crosslinking with luminescent centers^22,48–50^.

Recently, the scientific community has witnessed a convergence of studies on chirality and carbon dots^51^. Thus, the recent developments and major milestones on chiral carbon dots are in the focus of this review. The emergence of chiral carbon dots occurred quite recently, with the first reported examples appearing in 2016, when several groups introduced both top-down and bottom-up synthesized chiral nanoparticles^52–55^. Early studies were mostly devoted to synthesis, and some obvious applications such as enantiomeric recognition and selection. As the field of chiral carbon dots gained more traction, more systematic studies on the origin and mechanism of chirality surfaced^56–58^. Recent years have brought potential applications of chiral carbon dots into the spotlight, such as multi-color-emission^59^, chiral catalysis^60^, and bio-applications such as DNA rearrangement^61^. This was complemented by interesting optical phenomena of these materials, such as two-photon absorption^62^ and circularly polarized light emission^63,64^. Overall, chiral carbon dots attracted the scientific community due to the combination of low cytotoxicity, optical and chiroptical properties which are useful for bio-sensing, catalysis, and imaging.

This review aims to highlight all the important aspects of the novel field of chiral carbon dots, and is organized as follows. We first introduce chirality in general, and summarize experimental techniques which are used for chirality measurements. We then presents synthetic methods used for the fabrication of chiral carbon dots, and discusse their morphology, optical and chiroptical properties. In the last Chapter, some promising emerging applications of chiral carbon dots in sensing, bioimaging, and catalysis are considered. As a conclusion, we offer an outlook on this field, introducing perspectives and challenges of the future development of chiral carbon dots.

## Chirality: experimental techniques and molecular/nanoscale examples

**Fig. 1:**
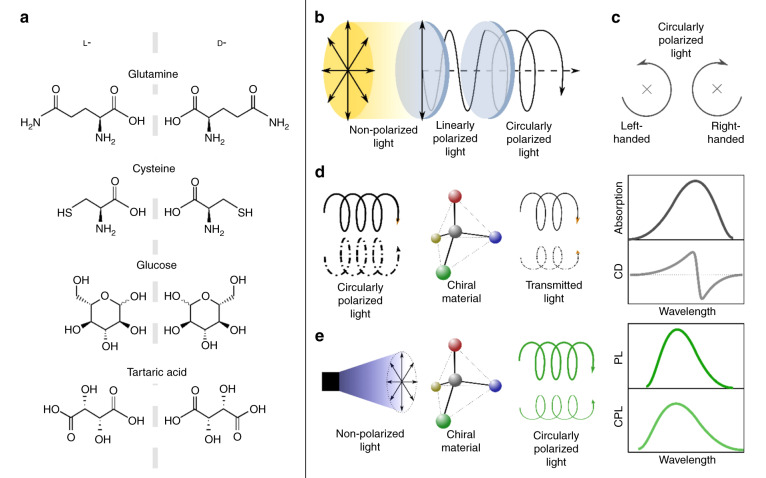
Optical and molecular principles of chirality and respective measurements. **a** Examples of chiral organic molecules which have been used in the synthesis of chiral carbon dots. **b** Illustration of nonpolarized, linearly polarized, and circularly polarized light. **c** Illustration of left- and right-handed circularly polarized light. **d** Schematic illustration of CD signal measurements, with idealized absorption and CD spectra shown on the right. **e** Schematic illustration of CPL signal measurement, with idealized PL and CPL spectra shown on the right

Chirality is a widespread phenomenon in nature, ranging from helical DNA’s and RNA’s enantiomeric components to the shape of galaxies^65^. Chiral organic compounds are an important part of the pharmaceutical industry. In molecular chemistry, chiral molecules (called enantiomers or optical isomers) are those which have the same chemical composition but constitute mirror images of each other and cannot be superimposed by any symmetry operations. Enantiomers have the same composition in terms of constituting atoms and functional groups, and thus possess identical physical and chemical properties except for their optical activity, which is defined as the ability to rotate plane-polarized light in opposite directions. Still, their interaction with biological objects may differ greatly, in terms of toxicity, metabolism, and therapeutic activities^66,67^. Several designations of enantiomers can be found in literature, such as D/L, R/S, and (+)/(−), which are based on Latin words *dexter*/*laevus* and *rectus*/*sinister*; those words are related to the ability of those molecules to rotate light clockwise (+) or counter-clockwise (−). In Fig. 1a, several examples of chiral molecules that have been used frequently for chiral carbon-dot synthesis^68–71^ are presented, such as L-/D-glutamine, L-/D-cysteine, L-/D-glucose, and L-/D-tartaric acid. It can be seen that the components of each of the sets of enantiomers are the same, just in a mirrored configuration. The optical response of intrinsic chiral molecules and nanomaterials can be described by the interplay of the induced electric dipole moment and the induced magnetic dipole moment. The interested reader is referred to refs. ^72–75^ for a deeper understanding of chiral light-matter interaction.

### Chiroptical spectroscopy

In this section, we provide a short summary of chiroptical spectroscopy methods; more detailed information can be found in recent topical reviews^76–78^. Circularly polarized light is an electromagnetic wave with a constant magnitude and its vector constantly rotating perpendicular to the propagation axis; it can be realized by passing a linearly polarized light through a quarter-wave plate with an optical axis at 45°, or a photoelastic modulator (Fig. 1b). Clockwise and counter-clockwise rotation in the direction of propagation is referred to as a right-handed and left-handed circularly polarized light, respectively (Fig. 1c). Light with one of those polarizations interacts in a different way with a chiral substance, which results in an inequality of left- and right-handed light absorption; this is referred to as circular dichroism (CD). Its value is defined as: $CD={A}_{L}-{A}_{R}$, with ${A}_{L}$ and ${A}_{R}$ being absorption of left- and right-handed polarized light, respectively. CD is further categorized into electronic circular dichroism (ECD) which occurs in the range of electronic transitions and is mostly observed in the ultraviolet (UV)-visible spectral range; and vibrational circular dichroism (VCD) corresponding to the vibrational energy levels which are measured in the infrared (IR) spectral region^77^. ECD and VCD have been extensively used to identify the chirality of materials, such as thin films^79^ or supramolecular structures^80^. Moreover, chirality can be also observed in the emission spectra, and in this case it is referred to as circularly polarized photoluminescence (CPL).

To avoid concentration-dependent artefacts in CD measurements, a dissymmetry factor ${g}_{abs}$ normalized by an average absorption (*A*) is introduced: ${g}_{abs}=\frac{CD}{A}=\frac{{A}_{L}\,-\,{A}_{R}}{\frac{({A}_{L}\,+\,{A}_{R})}{2}}$. From this definition, $|{g}_{abs}|\le 2$. For many materials, values of ${g}_{abs}$ are in the range of 10^−4^ to 10^−2^, with maximum values of 0.75 and 1 reported for thin films formed from enantio-pure prolinol functionalized squaraines^79^ and polyfluorene, respectively^81^. Many publications also report molar ellipticity, which is directly related to CD as: $[\theta ]=100\times \frac{ln10}{4}\frac{180}{\pi }\times CD=3298CD$.

A schematic for CD signal measurement is illustrated in Fig. 1d. Circularly polarized light of a determined wavelength passes through chiral material, which absorbs left- and right-handed circular polarized light differently; a CD signal versus wavelength of incident light can be calculated. Chiral nanoparticles containing two or more chromophores with matching energy levels display exciton coupling, the interaction of electrically allowed optical transitions. In this case, an ECD spectrum shows two extrema with opposite signs, which is called bisignate Cotton effect. The position of a zero ECD signal between these two extrema corresponds to the position of the absorption band maximum of the corresponding electronic transition, as in the first derivative of absorption spectra (Fig. 1d, right panel)^82,83^.

Circularly polarized luminescence is defined by the difference in intensities of left- and right-handed polarized emission: $CPL={I}_{L}-{I}_{R}$. Similar to the absorption, a dissymmetry factor of emission is introduced: ${g}_{PL}=\,\frac{2CPL}{I}=\frac{{I}_{L}-{I}_{R}}{\frac{({I}_{L}+{I}_{R})}{2}}$, with $|{g}_{PL}|\le 2$ and linear dependency on ${g}_{abs}$^84^. Fig. 1e illustrates the measurement of CPL: a CPL signal can be observed from a chiral and light-emitting sample following excitation by nonpolarized light. A quarter-wave plate or photoelastic modulator is used to convert circularly polarized light into two perpendicular linear polarizations, followed by polarization selection with a linear polarizer. CPL signal and ${g}_{PL}$can then be calculated with spectra measured using two perpendicular polarizer settings^85^. Peaks of CPL spectra and PL spectra often coincide (Fig. 1e, right panel).

According to Rosenfeld^86^, the quantum mechanical property connected to electronic circular dichroism is the rotational strength defined as $R=Im\overrightarrow{{{\boldsymbol{\mu }}}_{ij}}\cdot \overrightarrow{{{\boldsymbol{m}}}_{ij}}$, where $\overrightarrow{{{\boldsymbol{\mu }}}_{ij}}$ and ${{\boldsymbol{m}}}_{ij}$ are operators of electric and magnetic moment vectors, respectively. Both CD and CPL signals are proportional to the quantum mechanical dipole moment, which is given by: $D=\,{|\overrightarrow{{{\boldsymbol{\mu }}}_{ij}}|}^{2}+\,{|\overrightarrow{{{\boldsymbol{m}}}_{ij}}|}^{2}$. Dissymmetry factors ${g}_{abs}$ or ${g}_{PL}$ in an isotropic medium are given as: $g=\frac{4R}{D}=\frac{4|\overrightarrow{{{\boldsymbol{\mu }}}_{ij}}\cdot \overrightarrow{{{\boldsymbol{m}}}_{ij}}|\cdot \,\cos {\rm{\theta }}\,}{{|\overrightarrow{{{\boldsymbol{\mu }}}_{ij}}|}^{2}+\,{|\overrightarrow{{{\boldsymbol{m}}}_{ij}}|}^{2}}$ in the presence of a negligible electric quadrupole moment. g-factors depend on the magnitude and angles of rotational and dipole moments, and thus give insights into electronic transitions occurring in a chiral material. The information extracted from CD signals is related to electronic ground states, while CPL signals are useful for determining the configuration of excited states^84^.

### Chirality at nanoscale

Achievements of synthetic chemistry combined with the rapid development of nanotechnology opened up an opportunity to produce a wide range of artificial chiral objects at nanoscale^6,9,87,88^. Among them, chiral semiconductor quantum dots^89,90^, perovskite nanocrystals^91,92^, metal nanoparticles^93,94^ as well as chiral carbon dots and chiral graphene quantum dots^53,95^ have been reported. In these nanostructures, optical activity can be introduced via several pathways. Some materials exhibit an intrinsic chiral crystal structure, with a few examples, such as α-HgS^96^, quartz^97^, or metal nanocages^26,27,98^. Strain and the subsequent change of the crystal structure of nanoparticles can also cause the appearance of the optical activity in non-chiral materials^99^. By means of colloidal chemistry, nanoparticles can be synthesized in different shapes ranging from spherical dots and elongated nanorods to nanoplatelets, nanoscrolls, nanoribbons, and tetrapods; some of these shapes cause intrinsic optical activity^18,100,101^. Another way to introduce chirality is functionalization of the nanoparticle’s surface with chiral ligands, which may result in hybridization of their energy levels and/or distortion of their surface. Using this approach, plenty of chiral semiconductor nanoparticles have been reported, such as CdS^89^, CdSe^101,102^, and CdTe^103–105^ quantum dots, perovskites nanocrystals^91^, and nano-sheets^106^. Moreover, it was shown that chiral quantum dots can also be synthesized without using chiral precursors or ligands, yet enantiomer nanoparticles could be separated^90^, which pointed out that their optical activity may originate from chiral defects at the particle’s surface.

Chirality may also be caused by the nanoparticle’s assembly into a chiral structure. DNA-origami is one of the examples of chiral assembly, which endows the heterostructure with optical activity as was often reported for metal nanoparticles^93,98,107,108^. Formation of chiral assemblies can be triggered through illumination with circularly polarized light, as was shown for CdTe^109^, CdSe/ZnS^110^, and gold superstructures^108^. The use of chiral environments and/or templates can also induce the optical activity of formed nanostructures, as was demonstrated for metasurfaces and solvent-induced synthesis^111^.

To conclude, the chirality of nanostructures can be induced by several means: as an intrinsic chiral crystal structure, through chiral defects either in the core or at the nanoparticle’s surface, shape-induced and ligand-induced chirality, chiral assemblies, and environment-induced chirality. Most of these pathways can be implemented for carbon dots, as will be demonstrated in the next chapter.

## Synthesis of chiral carbon dots

In general, carbon dots can be synthesized either by top-down or bottom-up methods. Top-down methods are those which rely on cutting down macroscale materials to produce carbon nanoparticles^112–114^. Precursors can be graphene sheets^115^, carbon nanotubes^116^, and even candle soot^117^. Cutting can occur by chemical oxidation^118^, laser treatment^119,120^, hydrothermal^115^, or electrochemical methods^121,122^. Since most of the precursor materials used in those top-down approaches exhibit crystalline structures, carbon dots produced by these methods inherit the sp^2^-hybridization across the entire domain.

Bottom-up methods towards carbon dots rely on solution-based chemical synthesis, and involve polymerization and carbonization of molecular precursors^123^. They can be further subdivided according to the chemical nature of precursors used, namely non-conjugated or conjugated molecules^124^. Most common non-conjugated molecular precursors include combinations of citric acid with ammonia^125^, ethanolamine^126^, ethylenediamine, and its derivatives^41,127^, cysteamine^128^, L-cysteine^128^, urea, and thiourea^124,129^. Conjugated molecular precursors include phenylenediamines and their derivates^130^, and phenol and its derivatives^131^.

As noted, chirality in nanoparticles may originate from their intrinsic chiral crystal structures along with chiral defects, chiral shapes, ligand-induced energy structure, chiral assembly, and chiral environments. In the following, we will consider examples of synthetic pathways according to the abovementioned chirality origins, while still differentiating between the two major synthetic approaches — top-down and bottom-up.

### Top-down synthesis

First chiral carbon dots synthesized by top-down methods were reported almost simultaneously by Vázquez-Nakagawa et al.^54^. and Suzuki et al. in 2016^53^, who applied post-synthetic treatment of achiral carbon nanoparticles with chiral molecules. Vázquez-Nakagawa and co-workers started from graphene quantum dots produced by the method of Sekiya et al.^132^. Graphite was cut down in a solution of H_2_SO_4_ and HNO_3_ under ultrasonication, followed by heating at 120 °C for 24 h. After dialysis, carbon dots with an average diameter of 22 nm and narrow size distribution were separated by column chromatography, and treated with thionyl chloride, and then reacted with (*R-*) or (*S-*) 2-phenyl-1-propanol to form chiral nanoparticles. X-ray diffraction (XRD) confirmed the presence of (002) interlayer spacing of graphite; Fourier transform infrared (FTIR) spectroscopy showed stretching C–O–C vibrations of ester groups at around 1200 cm^−1^ and 1300 cm^−1^ which formed at the surface of carbon dots through passivation with chiral ligands.

**Fig. 2:**
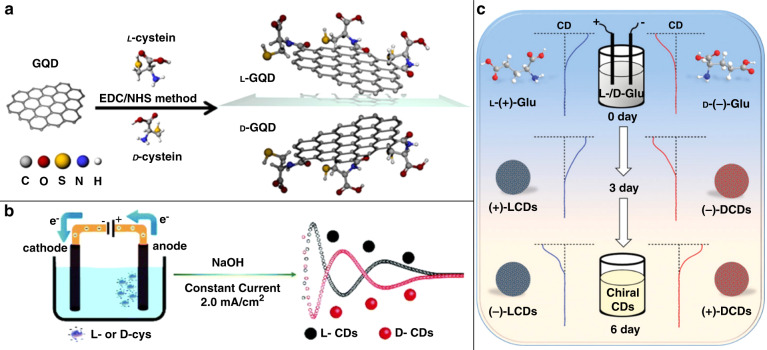
Examples of top-down synthesis of chiral carbon dots. **a** Surface treatment of graphene quantum dots with cysteine; **b** electrochemical exfoliation of graphite anode in the presence of L/D-cysteine; **c** electrochemical exfoliation of a graphite anode in the presence of L/D-glutamic acid. **a** Adapted with permission from^53^ Copyright 2016 American Chemical Society. **b** Adapted with permission from^135^ Copyright 2018 Royal Society of Chemistry. **c** Adapted with permission from^68^ Copyright 2019 Wiley-VCH GmbH

Suzuki and co-workers^53^ used a similar method of cutting down carbon precursors in a mixture of H_2_SO_4_ and HNO_3_, but started from pitch carbon fibers (the original method developed by Peng et al.^133^) instead of graphite. In their approach, cysteine has been used as a chiral ligand. Produced carbon dots were smaller in size (2–7 nm) and possessed the same (002) interlayer spacing as those synthesized from graphite^54^. Chiral D- and L-cysteine molecules were covalently bound to carbon dots’ surface by a EDC/NHS (EDC: N-(3-dimethylaminopropyl)-N′-ethylcarbodiimide hydrochloride; NHS: N-hydroxy succinimide) crosslinking method^134^ (Fig. 2a). FTIR spectroscopy confirmed covalent bonding of cysteine, namely S–H and C–N bonds at 2390 cm^−1^ and 930 cm^−1^, respectively.

Electrochemical techniques have also been used to fabricate chiral carbon dots^68,135^. Achiral graphene quantum dots were produced following the method of Ahirwar and co-workers^136^, who exfoliated graphene quantum dots from graphite rods in the presence of citric acid in NaOH solution. Employing this method, Hu et al. developed a one-pot top-down synthetic approach towards chiral carbon dots^135^. A NaOH solution containing L-cysteine or D-cysteine as chiral precursor was prepared, and graphite rods were immersed, serving as anodes and cathodes (Fig. 2b). A current of 0.02 A was applied for 72 h, resulting in exfoliation of the graphite anode; the solution turned brown and spherical carbon nanoparticles formed were purified by dialysis. These carbon dots had a diameter of 4–5 nm, and a lattice spacing of 0.21 nm belonging to the (100) graphite facet, stemming from the sp^2^ hybridization of the carbon core.

Zhang et al.^68^ applied a very similar electrochemical exfoliation approach, but used L- and D- glutamic acid instead of cysteine as a chiral precursor in NaOH solution (Fig. 2c). The obtained carbon dots were 3–7 nm in size, with ~7.5% nitrogen content as determined by X-ray photoelectron spectroscopy (XPS). Thus, the use of top-down synthetic methods mostly resulted in the formation of graphene quantum dots, whose chirality originated from the presence of chiral ligands on the achiral core’s surface.

### Bottom-up synthesis

Bottom-up synthesis of carbon dots denotes methods that produce luminescent carbon nanoparticles from molecular precursors via their pyrolysis during hydrothermal or microwave heating processes. Advantages of these methods are an easy, scalable, cost-effective synthesis, and a wide variety of precursors which can be used^137^. These precursors can also include chiral molecules, as will be discussed in this section.

#### Cysteine-based chiral carbon dots

Many chiral carbon dots reported up to date employed L-/D-cysteine as a chiral precursor, which was used either as a single precursor in water or in combination with citric acid and some other carbon sources. The first publication devoted to carbon dots synthesized from a racemic mixture of cysteine and citric acid by a hydrothermal method by Zhang and He^138^ in 2015 just focussed on their sulfur and nitrogen doping and did not address any optical (chiral) activity of nanoparticles. The same group followed up with a study that addressed their chirality in 2016^55^. In their synthesis, L- or D-cysteine were dissolved in deionized water along with citric acid, followed by hydrothermal treatment at 180 °C. Transmission electron microscopy (TEM) and atomic force microscopy (AFM) confirmed the size of carbon dots to be in the range of 2–5 nm. In another related study, Zhang et al. synthesized carbon dots via a hydrothermal procedure from L-/D-cysteine and citric acid as well, and reported slightly larger sizes of about 4–8 nm^139^.

The synthesis of chiral carbon dots by a one-pot approach employing only L-/D-cysteine (without any other precursors) in sodium hydroxide solution performed by hydrothermal methods has been reported by several groups^61,69,140,141^. Hu et al.^69^. and Liu et al.^141^. both synthesized chiral carbon dots from L-/D-cysteine in a NaOH solution under hydrothermal treatment at 120 °C for 16 h. However, Hu reported chiral carbon dots with a diameter of 4–5 nm, using 0.06 g NaOH in 15 mL of water, while Liu reported a much smaller diameter of just 1.25 nm in a solution of 1 g NaOH in 10 mL of water. Thus, the pH value in this synthesis might have a strong influence on the size of produced carbon dots.

Branzi et al. studied the formation mechanism of cysteine-based chiral carbon dots produced at room temperature in a reaction catalyzed by copper(II) salts^95^. The formation process of chiral carbon dots was claimed to be influenced by several features of cysteine molecules: free thiol groups took part in redox reactions and resulted in the formation of radical species; amine and carboxylic functional groups enhanced stability of the oxidized state; and hydrogen atoms assisted in radical hydrogen transfer reactions forming carbon-centered radicals. Other organic molecules, such as glycine, thioglycolic acid, methionine, penicillamine, and cysteamine were studied as well, but did not result in the formation of carbon dots at similar reaction conditions.

Other synthetic approaches towards chiral carbon dots utilizing L-/D-cysteine and citric acid were pyrolysis^142^ and microwave-assisted^57^ methods. Victoria et al. demonstrated the formation of 5-oxo-3,5-dihydro-2H-thiazolo[3,2-a]pyridine-3,7-dicarboxylic acid (TPDCA) during microwave irradiation of a citric acid and cysteine mixture, which was further polymerized and carbonized to form chiral carbon dots^57^. XRD measurements showed their semi-crystalline structure with (002) planes of graphene in between amorphous regions. Contrary to the top-down produced carbon dots passivated with cysteine, these chiral carbon dots did not exhibit a S–H bond in the FTIR spectra. Das et al. used urea in their hydrothermal synthesis from L-/D-cysteine and citric acid and produced carbon dots of ~5 nm in size, similar to those synthesized without cysteine molecules^62^.

Besides citric acid, which has been most commonly used as a carbon co-source for chiral carbon dots synthesized from L-/D-cysteine, a number of other achiral precursors have been reported, such as ethylene diamine^63,143^, Jeffamine ® ED-900^144,145^, sucrose^146,147^, arginine^58^, and o-phenylenediamine^59^. While all the abovementioned methods used water as a solvent, Arshad et al. synthesized chiral carbon dots from cysteine and p-benzoquinone in ethanol by microwave treatment^148^. The resulting dots were smaller (2.7 nm in diameter) and highly uniform. Their graphitic structure was revealed by TEM, by observation of (002) lattice planes. The elemental analysis showed doping with 9% nitrogen and 6% sulfur content, while FTIR spectra showed the presence of N–H and S–H bonds at 3376 cm^−1^ and 2682 cm^−1^.

Bottom-up synthesized achiral carbon dots may become optically active via post-synthetic surface treatment, which can be referred to as ligand-induced chirality. Das et al. developed a room-temperature post-synthetic treatment of achiral carbon dots with EDC/NHS for bonding with L/D-cysteine. The resulting carbon dots had a larger mean size of 7 nm compared to 5 nm for achiral carbon dots^62^.

#### Other chiral precursors

While L- and D-cysteine were most frequently used as chiral precursors for chiral carbon dots, plenty of other chiral molecules has been explored as well. Among those were amino acids, such as aspartic acid^149,150^, glutamic acid and methionine^151^, lysine^144,145^, alanine^150^, proline^152^, tryptophan^56,59^, and tyrosine^60^, as well as other organic compounds such as guanosine 5′-monophosphate^52^, tartaric acid^153^, sparteine^146^, glucose^71^ and glucosamine^150^, glutathione^143^, ascorbic acid^143^, pencillamine^153^, and (R,R)/(S,S)-1,2-cyclohexanediamine^58^.

**Fig. 3:**
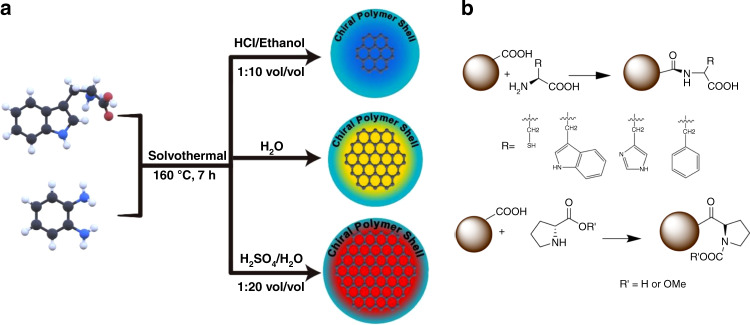
Examples of bottom-up synthesis of chiral carbon dots. **a** Solvothermal synthesis from L-/D-tryptophan and phenylenediamine; **b** conjugation of amino acids at the surface of pre-synthesized CDs. **a** Adapted with permission from^59^ Copyright 2021 Wiley-VCH GmbH. **b** Adapted with permission from^147^ Copyright 2018 American Chemical Society

The first synthetic method for chiral carbon dots without the use of cysteine was reported in 2016 by Vulugundam et al., who applied microwave treatment to sucrose and either (+)- or (−)-sparteine to produce rather large spherical carbon nanoparticles of 22 nm diameter^146^. More recently, Ru et al. synthesized chiral carbon dots from L-/D-tryptophan and phenylenediamine solvothermally in different solvents, which altered the size and luminescence of these nanoparticles^59^ (Fig. 3a). Other combinations of chiral and achiral precursors included L-glutathione and citric acid^143^, L-/D-lysine and Jeffamine ED-900^144,145^, and (R,R)/(S,S)-1,2-cyclohexanediamine with arginine^58^. Additionally, some synthetic methods without the use of solvents have been proposed, such as heating of methionine, glucose, glucosamine, aspartic acid or alanine to a temperature above their melting point^150^. as well as D-tartaric acid with L-penicillamine and vice versa^153^.

Pursuing the concept of ligand-induced chirality, Ostadhossein et al. performed surface treatment of carbon dots via EDC/NHS coupling to introduce their chirality with a plethora of chiral amino acid (proline, phenylalanine, histidine, tryptophan, alanine, and proline methyl ester) and sucrose in an aqueous solution (Fig. 3b). This process appeared to be universal for a wide range of amino acids and led to structurally and optically similar carbon-dot materials^147^.

### Chiral composites comprising carbon dots

Retaining chirality while fabricating composite materials based on chiral carbon dots for their further applications is still challenging. In several instances, different matrices have been employed, whereas carbon dots were introduced during a matrix formation by different means.

**Fig. 4:**
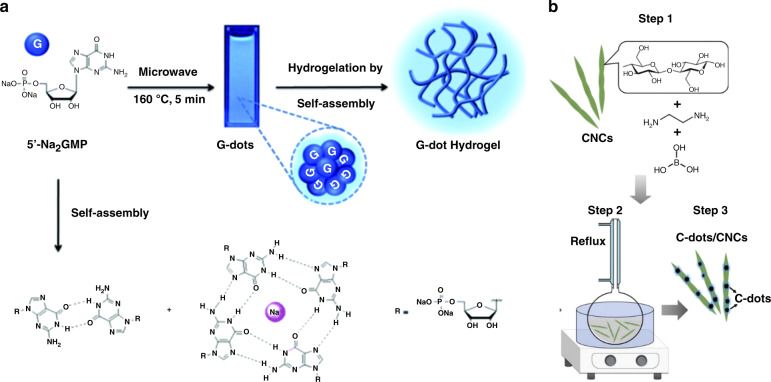
Examples of chiral heterostructures with carbon dots. **a** Formation of guanosine 5’-monophosphate (5′-GMP) carbon dots and the carbon dots-based hydrogel; **b** synthesis of chiral carbon dots at the surface of cellulose nanocrystals. **a** Adapted with permission from^52^ Copyright 2016 Royal Society of Chemistry. **b** Adapted with permission from^63^ Copyright 2019 Wiley-VCH GmbH

Ghosh et al. reported the synthesis of chiral carbon dots from guanosine 5′-monophosphate (5-GMP). In an aqueous solution, these molecules tend to form hydrogels (Fig. 4a), while microwave irradiation of such a solution produced superstructures of carbon dots that retained the chirality^52^. Zhou et al. doped a N,N′-bis(octadecyl)-D-aminoglutamic diamide (DGAm) gel with chiral carbon dots to enhance the emission signal of the resulting fluorescent films; the chiral properties of the gel and the chiral carbon dots were superimposed^151^. Ru et al. reported a co-gel of DGAm/LGAm and chiral carbon dots, with circularly polarized emission induced in this composite structure^59^. Another example of a matrix, which is suitable to retain intrinsic chirality of carbon dots, is metal-organic frameworks (MOF), such as demonstrated by Liu et al. who embedded chiral carbon dots synthesized from L-/D-glucose and sodium dihydrogen phosphate into Ni-based MOF^71^.

Chekini et al. synthesized carbon dots at the surface of cellulose nanocrystals, where the latter served as a carbon source and induced chiral properties at the same time (Fig. 4b)^63^. Similarly, Zheng et al. also reported cellulose nanocrystals to induce chirality onto helical superstructures, but instead of exploiting carbon within the cellulose, carbon dots and cellulose were co-assembled during the evaporation of solvent^64^.

To summarize this section, chirality in carbon dots can be induced via the following methods: (i) one-pot synthesis resulting in a formation of a chiral carbon core; (ii) surface treatment with chiral molecules leading to ligand-induced chiral signal; (iii) assembly or asymmetric synthesis at chiral surfaces resulted in carbon-dot-based composites.

## Properties of chiral carbon dots

While chiral carbon dots have been reported to possess similar properties to classical carbon dots in terms of their shape, size, structure, and emission, they also showed distinct chiroptical features. In the following sections, we will consider different properties of chiral carbon dots as compared to achiral ones.

### Morphology: size, shape, and structure

The majority of reported chiral carbon dots had spherical shapes with rather widely varying diameters, ranging from 2 nm^53,55,63,144^ to 22 nm^54^. Carbon dots synthesized using top-down methods often exhibited crystalline cores, as their precursors were graphite or graphene-based materials. Suzuki et al. synthesized chiral carbon dots through exfoliation of carbon fibers and passivation with cysteine^53^. XRD revealed their crystalline graphene structure with a peak at 2*θ* = 25°, attributed to (002) carbon-to-carbon spacing of 3.7 Å independent of chiral modifications^53^. A similar structure was reported by Vazquez-Nakagawa et al. who used graphite as a carbon source to produce chiral carbon dots through exfoliation. XRD revealed their graphite pattern with a (002) interlayer spacing of 0.338 nm^54^. Several examples of bottom-up synthesized chiral carbon dots with the crystalline structure of carbon cores have been reported as well. Ru et al.^59^ synthesized chiral carbon dots from L-/D-tryptophan and phenylenediamine; both of those precursors possess conjugated structures that led to the appearance of sp^2^ domains. This was confirmed by TEM images which revealed lattice fringes of 0.21 nm attributed to in-plane lattice distance in (100) graphene. The variation in size of sp^2^-conjugated domains resulted in a change in the optical properties of carbon dots^59^. Likewise, several other studies reported bottom-up synthesized carbon dots with graphene/graphite domains^56,61,62,69,135,140,152,153^.

At the same time, there are numerous reports on bottom-up synthesized chiral carbon dots which did not possess a distinct crystal structure and appeared to be mostly amorphous. Ghosh et al. synthesized chiral carbon dots from guanosine 5’-monophosphate and revealed disordered carbon in their cores by Raman spectroscopy^52^, which led the authors to conclude that their emission likely originated from surface states or molecular fluorophores. Victoria et al.^57^ synthesized chiral carbon dots from cysteine and citric acid which exhibited a broad XRD peak (halo) at about 17° which generally indicates an amorphous structure. However, they also showed several sharp XRD peaks at 21°, 30°, 31°, 33°, and 35° associated with graphene, which possibly originated from crystalline subdomains within the amorphous structure^52^. Zeng et al. and Deka et al. reported amorphous carbon dots with a size of 2–5 nm synthesized by a bottom-up method from various chiral amino acids or other chiral organic materials^143,150^.

### Absorption and photoluminescence

Absorption and photoluminescence (PL) of chiral carbon dots in general resembles those of achiral carbon dots obtained by similar synthesis methods. Reported absorption spectra often exhibited bands at 240–270 nm, which can be ascribed to π–π* transitions in the sp^2^-hybridized carbon core^58,69,140^. Another recurring absorption peak was commonly reported in the range of 300–350 nm, and was attributed to n–π* transitions or carboxyl groups^57,140^. Ru et al. reported absorption bands for yellow and red-emitting chiral carbon dots located at 425 and 535 nm, respectively, which were attributed to the transitions related to surface states^59^.

A lot of chiral carbon dots exhibited violet or blue emission in the range of 410–460 nm. Chiral carbon dots synthesized from citric acid usually fall in this range. Das et al. synthesized chiral carbon dots from L/D-cysteine, citric acid, and urea with an emission at 450 nm^62^, while others performed their syntheses from L/D-cysteine and citric acid without urea, and reported PL peaks between 418 and 440 nm^55,57,138,139,142^. Chiral carbon dots synthesized from citric acid combined with aspartic acid^149^ or proline^152^ also showed a PL peak at 420 nm, while the use of tyrosine as a chiral precursor yielded a slightly longer emission wavelength of 450 nm^60^. Chiral carbon dots synthesized in NaOH solution from a single chiral precursor without any additional carbon or dopant sources tend to have slightly longer emission wavelengths, such as 460 nm to 510 nm for cysteine^61,69,135,140^, 476 nm for tryptophan^56^, and 445 nm for glutamic acid^151^. Besides these two common approaches, other reports were focused on chiral carbon dots emitting at longer wavelengths. Arshad et al. synthesized chiral carbon dots in an ethanolic solution of para-benzoquinone and L-cysteine, which emitted at 445 nm dispersed in methanol, while the PL peak appeared at 546 nm when they were dispersed in water^148^. Ru et al. demonstrated that the emission of chiral carbon dots produced from L-/D-tryptophan and o-phenylenediamine depends on the reaction solvent: for a 1:10 mixture of HCl and ethanol their emission peak was observed at 441 nm, for an aqueous solution at 546 nm, and for a 1:20 mixture of H_2_SO_4_ and water at 604 nm^59^, because highly acidic environment allowed the formation of larger conjugated domains with a narrower bandgap. Vulugundam et al. synthesized chiral carbon dots from sucrose and (+)/(−)-sparteine in water, which exhibited a very broad emission band in the range between 450 and 600 nm^146^. Chiral carbon dots synthesized using a top-down approach and EDC/NHS passivation with L-/D-cysteine or (S)/(R)-2-phenyl-1-propanol as chiral precursors as reported by Vazquez-Nakagawa^54^ and Suzuki^53^, emitted in the yellow spectral range at 520–550 nm.

Reported photoluminescence quantum yields (PLQY) for chiral carbon dots covered a broad range from 12%^135^ to 68%^142^, with an average value of 30–40%. Li et al. reported that PLQY increased from 4.1% for carbon dots synthesized from citric acid only to 68% for those produced with the addition of cysteine. Amino acids such as cysteine were shown to be useful for PLQY improvement^154^. We recall that sulfur was also shown to improve PLQY of carbon dots via doping, and also enabled longer-wavelength emission^155,156^.

In terms of PL decay and the related PL lifetimes, typical values for chiral carbon dots were reported in the range of 3 and 11 ns. Đordevic et al.^58^ reported chiral carbon dots synthesized from arginine and cyclohexanediamine with a double-exponential decay (slow component τ_1_ = 11 ns and fast component τ_2_ = 1.2 ns) and an average PL lifetime of 8 ns. Ru et al.^59^ reported a slow component τ_1_ = 6 ns and fast component τ_2_ = 2 ns for carbon dots produced from L-/D-tryptophan and o-phenylenediamine in different solvents, which were assigned to recombination through core and surface states, respectively. We notice that average PL lifetimes for carbon dots produced from the same precursors may vary, and carbon dots made from citric acid and cysteine were reported to exhibit values between 4^62^ and 11 ns^138^.

### Chiroptical properties

**Fig. 5:**
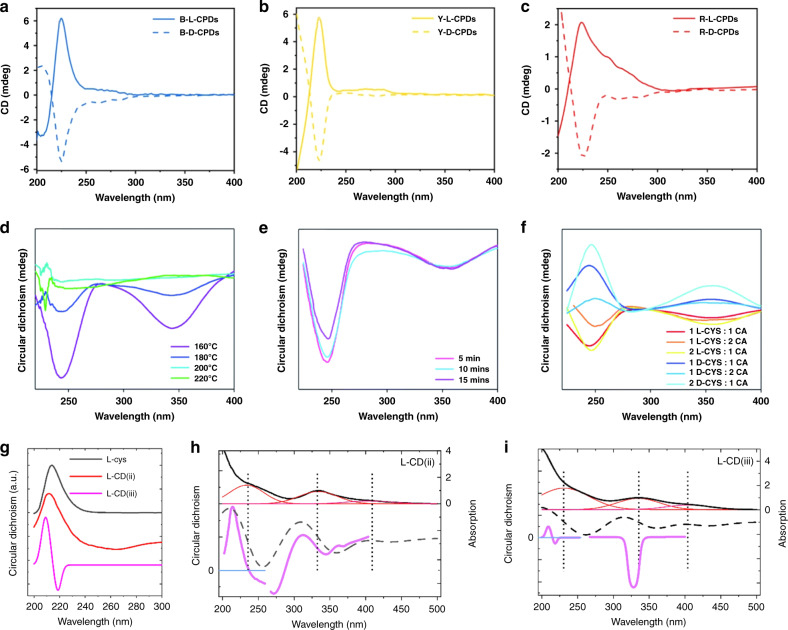
Chiroptical properties of chiral carbon dots. **a–c** CD spectra of carbon dots synthesized from L-/D-tryptophan and phenylene diamine in different solvents, with **a** blue, **b** yellow, and **c** red emission. **d–f** CD spectra of chiral carbon dots synthesized under different synthetic conditions: **d** reaction temperatures, **e** synthesis time, **f** precursor ratios between L-/D-cysteine and citric acid. **g–i** CD spectra of carbon dots synthesized from citric acid, urea, and L-cysteine by surface treatment (the sample called L-CD (ii)), and one-pot carbonization (sample L-CD (iii)): (g) Comparison of CD spectra of L-cysteine (L-cys, black line), L-CD (ii) (red line), and L-CD (iii) (pink line); **h, i** Absorption (black and red lines), its first derivative (black dashed lines) and circular dichroism spectra (pink lines) of L-CD (ii) (**h**) and L-CD (iii) (**i**). **a–c** Adapted with permission from^59^ Copyright 2021 Wiley-VCH GmbH. **d–f** Adapted with permission from^57^ Copyright 2020 Royal Society of Chemistry. **g–i** Adapted with permission from^62^ Copyright 2021 Royal Society of Chemistry

Among chiroptical properties, circular dichroism (CD), including electronic circular dichroism (ECD) and vibrational circular dichroism (VCD), and circularly polarized luminescence (CPL) have been reported for chiral carbon dots. Suzuki et al.^53^ emphasized a high dissymmetric factor ${g}_{abs}$ of chiral carbon dots in the range of 210–220 nm, which originated from their chiral precursor L-/D-cysteine. Additionally, a weaker signal was detected in the range of 250–260 nm, which was only present in the CD spectra of chiral carbon dots but not of their chiral precursors^53^. This signal was attributed to the induced chirality via hybridization of electronic levels of chiral precursors with those of carbon dots. In the follow-up studies, a high-energy CD signal in the range of 200–240 nm inherited from the chiral precursors, plus a less intense signal at about 250–300 nm was frequently reported for chiral carbon dots. As an example, Ru et al.^59^ reported chiral carbon dots synthesized from L-/D-tryptophan, which emitted in three different colors depending on the solvent, whose CD spectra are given in Fig. 5a–c. A strong signal from the chiral precursor in the range of 200–240 nm was present for all three samples, together with a weak, asymmetric signal appearing in the range of 245–300 nm. The authors attributed this signal to π–π* transitions of aromatic sp^2^-domains enhanced by the chiral environment, which differed in spectral position for carbon dots synthesized in different solvents^59^. The CD signals of samples emitting at longer wavelength appeared to have a tail extending to longer wavelengths, indicating their stronger carbonization. Ðorđević et al. reported CD signals at 260 nm and 320 nm for their carbon dots, in accordance with their absorption spectra they were attributed to C=C and C=O transitions^58^.

Chiroptical properties of carbon dots are strongly dependent on synthesis parameters, as was shown by Victoria et al.^57^. Their chiral carbon dots were synthesized by a one-pot approach from citric acid and L-/D-cysteine in water at temperatures varying from 160 to 220 °C. They showed CD bands at around 240 nm and 345 nm (Fig. 5d), which coincided with positions of their absorption bands, but were not present in pure L-/D-cysteine, which peaked at 215 nm (Fig. 5g, black line), or achiral carbon dots. As the synthesis temperature increased from 160 to 220 °C, the intensity of the CD signal decreased, which was explained by a higher degree of carbonization, which broke up the chiral structure of cysteine ligands and incorporated them into the carbon core. This observation was supported by a reduced number of free thiols present at the surface, indicating the decomposition of fluorophores based on cysteine. An impact of prolonged synthesis time on the chirality of carbon dots has been reported in the same study as well: longer synthesis time resulted in a more hybridized carbon structure and decomposition of some chiral fluorophores at the same time, causing some reduction of CD signal (Fig. 5e). Figure 5f shows that the synthesis of chiral carbon dots from either L- or D-cysteine indeed resulted in formation of both enantiomers as can be seen from opposite signs of the CD signal, as well as an increased ratio of the chiral precursor (cysteine) to citric acid precursor leading to stronger signals. Branzi et al. studied the influence of the reaction time on the chiral signal of carbon dots synthesized at room temperature in a copper-catalyzed process^95^. Peaks in CD spectra were observed at 220 and 250 nm, whose intensity reached their maximum after 2 h of synthesis, followed by a gradual decrease.

Das et al.^62^ compared the optical activity of chiral carbon dots synthesized from citric acid, urea, and L/D-cysteine using either post-preparative surface treatment with chiral molecules or via one-pot synthetic approaches. Figure 5g provides a comparison of CD spectra of L-cysteine (black line) and carbon dots synthesized by surface treatment (red line) and one-pot (pink line) approaches, which were denoted by the authors as L-CD(ii) and L-CD(iii), respectively. A strong peak from L-cysteine at 210 nm was inherited by carbon dots produced via surface treatment with this molecule, alongside the rise of an additional broad peak at 240–280 nm, which was attributed to cysteine aggregates. For chiral carbon dots obtained by one-pot carbonization, two peaks in the UV-region at 209 and 219 nm were observed which were attributed to a Cotton effect caused by the hybridization of the high-energy electronic state of carbon dots with the cysteine energy level. Other peaks for both one-pot synthesis and post-preparative treatment were observed in the spectral region of absorption bands of n-π* transitions in the range of 350 nm and were attributed to the formation of a chiral carbon core.

The influence of chiral precursors on chiroptical properties of carbon dots synthesized via post-synthetic surface treatment was also studied by Ostadhossein et al.^147^ on examples of carbon dots with chiral cyclic acids attached to their surface *via* EDC/NHS conjugation. While chiral carbon dots functionalized with L-/D-alanine, L-/D-cysteine, L-/D-tryptophan, L-/D-phenylalanine, and L-/D-histidine exhibited a CD signal with the same sign as that of respective amino acids, those functionalized with L-/D-proline and L-/D-proline methyl ester showed inverse CD signals as compared to that amino acids^147^. It was emphasized that such an inversion of CD signal corresponded to amino acids containing a secondary amine. Moreover, on the example of proline it has been shown that a sign inversion did not originate from the aggregation of chiral molecules. Such a reversion of chirality was observed by Zhang et al. as well. They produced chiral carbon dots via an electrochemical method from L-/D-glutamic acid^68^. At the beginning of the reaction, chiral carbon dots with a similar chirality to glutamic acid were observed, while after 3 days of reaction the signal mostly vanished. However, for prolonged reactions of over 5 days the CD signal reappeared and switched signs^68^.

Another interesting manifestation of the optical activity of carbon dots is the chirality in the vibrational spectra. Ðorđević et al. reported VCD signal with strong bands at about 1600 cm^−1^ and 1350 cm^−1^, which were attributed to amine N–H and C–H bending vibrational modes^58^.

**Fig. 6:**
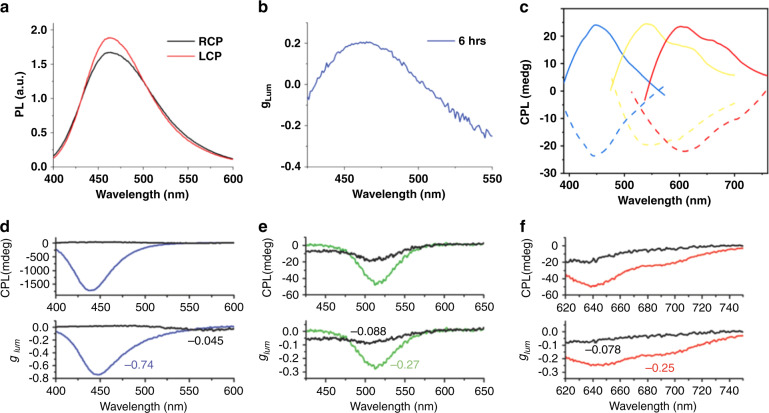
CPL in chiral carbon dots. **a** PL spectra of carbon-dot/cellulose nanocrystals, recorded using left- and right-handed circular polarizers (LCP and RCP, respectively) and **b** their g_Lum_ plotted as a function of emission wavelength. **c** CPL spectra of superstructures of blue, yellow, and red chiral carbon dots and glutamic diamide (solid and dotted lines show spectra of enantiomers). **d–f** CPL spectra and g_PL_ of cellulose nanocrystals and blue (**d**), green (**e**), and red (**f**) carbon dots from the carbon-dot/cellulose nanocrystal composites. Colored lines show photonic band gap superimposed on emission band; black lines show photonic band gap not overlapping with emission band. **a, b** Adapted with permission from^63^ Copyright 2019 Wiley-VCH GmbH. **c** Adapted with permission from^59^ Copyright 2021 Wiley-VCH GmbH. **d–f** Adapted with permission from^64^ Copyright 2018 Wiley-VCH GmbH

Besides the circular dichroism in absorption, circularly polarized light emission has also been demonstrated in chiral nanoparticles^157,158^, quantum dots^159^, and perovskite nanocrystals^160^. While a few reports mentioned a zero CPL for chiral carbon dots^53,58^, a CPL signal has been observed for their composites with other materials. Thus, carbon dots produced at the surface of cellulose nanocrystals showed CPL with ${g}_{PL}$ reaching 0.2 at the emission peak of 460 nm^63^ (Fig. 6a,b). Ru et al. synthesized superstructures from chiral carbon dots and L-/D-glutamic acid which showed a peak in CPL spectra at the same wavelength as the PL peak, namely, at 450, 560, and 610 nm^59^ (Fig. 6c). Zheng et al.^64^ fabricated thin films from the mixtures of blue/yellow/red carbon dots and cellulose nanocrystals, which showed photonic bandgaps tunable by salt added to the mixture. Figure 6d–f show CPL and ${g}_{PL}$ spectra of films produced from three different emissive carbon dots. In case of the superimposed photonic bandgap and PL band, ${g}_{PL}$ reached −0.74, −0.27, and −0.25 for blue, green, and red carbon dots-based films, respectively^64^.

### Two-photon absorption

Nonlinear optics describes effects and phenomena that exhibit a nonlinear dependence on parameters of excitation light. Nonlinear effects have been exploited for different applications, for instance, in data processing^161^ and microscopy^162^. The simplest nonlinear process is the second harmonic generation (SHG), in which two low-energy photons are absorbed and a single photon at double the energy is being emitted. For SHG to occur, non-centrosymmetry of the active materials is required^163^, and non-centrosymmetric achiral materials have been proposed to generate the second harmonics^164^. Since chiral materials are intrinsically non-centrosymmetric, they could be excellent candidates for two-photon absorption and subsequent frequency doubling. Zhang et al. and Das et al. reported two-photon absorption for chiral carbon dots synthesized from L-/D-cysteine and citric acid^62,138^. An indicator for two-photon absorption is the relation between integrated excitation and emission intensity which should be quadratic for second-order nonlinearities^165^. Das et al. irradiated chiral carbon dots at a wavelength of 800 nm, at which they emitted at 540 nm wavelength almost coinciding with their emission excited at 480 nm. Their integrated PL intensity (${I}_{PL}$) followed the power-law dependence on excitation intensity (${I}_{ex}$):${I}_{PL} \sim {I}_{ex}^{K}$ with *K* = 2.2, indicating an almost quadratic dependence^62^. Zhang et al. reported emission of around 418 nm at excitation in the range of 720–800 nm, with a quadratic power dependence of ${I}_{PL}$. Due to the excitation independence of emission of those carbon dots, the authors attributed their two-photon absorption to the lowest singlet state transitions^138^.

## Applications

### Applications of chiroptical nanomaterials

To illustrate potential areas of applications for chiral carbon dots, applications of chiroptical nanomaterials in general will be shortly introduced at the beginning of this chapter. Since the chiral ligands or chiral surface of chiral nanoparticles tend to interact selectively with different enantiomers and thus may result in a change in optical properties of the analyte, either in fluorescence or in circular dichroism, one of the promising applications of chiral nanoparticles is in sensing. For example, the interaction of Cd-based quantum dots capped with chiral ligands with different amino acid enantiomers resulted in a change of both the intensity^166,167^ and spectral position^168^ of their PL, as well as their CD signal^169^. Chiral nanoparticles have been used for drug sensing, such as ibuprofen, ketoprofen, aryl propionic acids, flurbiprofen, and naproxen^170^, and for sensing metal ions, such as Ni^2+^, Co^2+^ ^171^, and Pb^2+^ ^172^.

In another area of application, chiral nanoparticles served as a chiral catalyst, for instance, in direct asymmetric aldol reactions^173^. Genome editing is seen as one of the most promising new developments in therapeutics^174^. Methods such as CRISPR-Cas9 found great resonance in the scientific community^175^, resulting in high interest to find more efficient ways for DNA cleavage, which could be accomplished with chiral semiconductor nanocrystals^176^. In light of aforementioned applications and the fact that chirality has an influence on cytotoxicity of nanoparticles^177^, carbon dots with their intrinsic low cytotoxicity^178^ may be a good candidate for replacing Cd/Pb-based quantum dots in bio-applications such as gene editing.

Apart from bio-applications, circularly polarized light is used in polarizers for displays^179,180^. However, circular polarization usually requires expensive and bulky optical equipment, a challenge that might be solved using optically active nanomaterials. For example, control of CPL has been achieved in hybrid organic-inorganic perovskites by applying a magnetic field^181^.

### Applications of chiral carbon dots

As chiral carbon dots are non-toxic and can be synthesized by plethora of cost-effective and simple synthetic pathways, they may be an excellent candidate for sensing, imaging, and medical treatment.

#### Sensing

Chiral carbon dots capped with (+)/(−)-sparteine were used to selectively sense L- or D-cysteine^146^. In sight, aqueous solutions of L-cysteine or D-cysteine were prepared and their circular dichroism spectra measured. Afterwards, (+) or (−)-sparteine capped chiral carbon dots were added to those solutions at a fixed concentration of 0.5 mg/mL and stirred for 12 h. Through centrifugation, free chiral carbon dots were discarded, and a change of their circular dichroism recorded, indicating adsorption of L-/D-cysteine on the chiral carbon nanoparticles. Carbon dots capped with (−)-sparteine adsorbed 33% of L-cysteine, but only 14% of D-cysteine. As such, an enantiomeric excess of 19% of L-cysteine can be calculated. On the contrary, an enantiomeric excess of 15% was reported for D-cysteine with respect to (+)-sparteine. Along with cysteine, proline was checked as a probe leading to similar results, which showed a potential for broader applications for sensing of different amino acids.

**Fig. 7:**
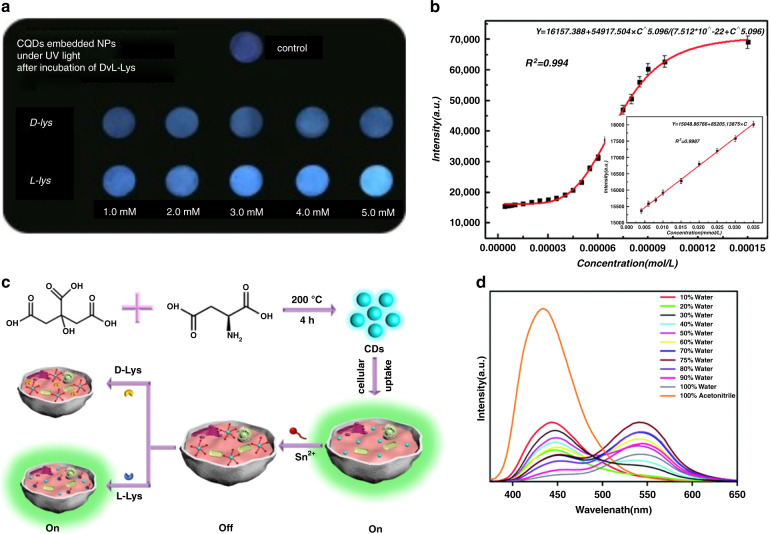
Examples of sensing applications of chiral carbon dots. **a** Enantioselective sensor for L-/D-lysine; **b** Sensor for L-/D-arginine in nonlinear and linear (inset) concentration ranges; **c** On-Off-On sensor for Sn^2+^ ions using L-/D-lysine capped carbons dots; **d** Moisture sensor. **a** Adapted with permission from^182^ Copyright 2019 Elsevier. **b** Adapted with permission from^143^ Copyright 2017 Royal Society of Chemistry. **c** Adapted with permission from^149^ Copyright 2020 Elsevier. **d** Adapted with permission from^148^ Copyright 2019 Royal Society of Chemistry

Copur et al. synthesized chiral carbon dots from L-cysteine as chiral precursor^182^. In a solution with L-lysine, their fluorescence intensity linearly increased with the concentration of L-lysine, which was attributed to a restriction of intramolecular vibrations and rotations reducing nonradiative relaxation^182^. On the other hand, D-lysine did not change the intensity of carbon-dot’s PL in such a way, and thus L-lysine was able to be sensed with a high degree of accuracy as well as the ratios between D-lysine and L-lysine in their enantiomeric mixtures. As a proof of principle, the authors demonstrated a paper sensor for the detection of L-lysine (Fig. 7a).

In another example, the enantiomeric selection was proven in electrochemical measurements, using an electrode made from graphite powder and L- or D-cysteine capped chiral carbon dots designated as L/D-chiral carbon-dot (CCD) electrode^69^. As shown by electrochemical impedance spectroscopy and linear sweep voltammograms, the electrode had a different pattern of response for L- or D-tartaric acid. The current of L-CCD electrode was about 30% higher in the presence of L-tartaric acid than in the presence of D-tartaric acid. Likewise, the D-CCD electrode had a 18% higher current in the presence of D-tartaric acid than with L-tartaric acid. Enantioselective recognition of L- and D-tryptophan has been shown by L- or D-tartaric acid capped graphene quantum dots^70^ in an electrochemical measurement. L-tartaric acid capped graphene quantum dots showed a higher current in the presence of D-tryptophan as compared to L-tryptophan, and vice versa for D-tartaric acid capped ones.

Zeng et al.^143^ presented a method to sense L-/D-arginine with chiral carbon dots which were produced using L-glutathione. An addition of L-/D-arginine enhanced their emission, but the concentration dependence was linear only at low concentrations of up to 10^–5^ mol/L (Fig. 7b). Also, L-arginine and D-arginine caused the same degree of PL improvement, making enantioselective sensing not viable. Other amino acids, metal ions, and inorganic anions were tested as well and did not have a measurable influence on PL. The increased PL signal was attributed to the prevention of electron-hole nonradiative recombination in the presence of arginine.

Besides reports on enantioselective sensing of amino acids with chiral carbon dots, metal ion sensing has also been widely reported. Ma et al. used chiral carbon dots produced via a hydrothermal method from L-/D-glutamine and citric acid to sense Fe^3+^ ions in solution, which quenched their PL by up to 80%^183^. The minimum detection limit has been reported as 0.014 mmol/L for L-glutamine and 0.011 mmol/L for D-glutamine-based carbon dots. A transfer of electrons occurred during interactions of chiral carbon dots with iron ions, which led to higher nonradiative recombination and thus quenched PL in this case. In the study by Gao et al., the addition of Sn^2+^ ions to a solution of L-aspartic acid capped chiral carbon dots significantly quenched PL signal as well, with a linear dependence on concentration and a detection limit of 0.057 μM^149^. The authors also demonstrated an On-Off-On sensor for Sn^2+^ ions using chiral carbon dots and L-lysine: the addition of L-lysine was claimed to remove bonds between Sn^2+^ and chiral carbon dots resulting in a reappearance of PL signal (Fig. 7c). No PL reappearance took place upon the addition of D-lysine, showing that this method is enantioselective indeed.

Arshad et al. developed a moisture sensing method based on L-cysteine capped chiral carbon dots^148^. PL of these carbon dots at 440 nm was quenched while another emission at 546 nm appeared in contact with water (Fig. 7d). The authors attributed this effect to aggregation-induced emission and used it to further develop a moisture sensing method for household products. A solution of chiral carbon dots in acetone was prepared, and a fixed amount of product was added, such as aspirin tablets, dried peas, etc. The water content in the product then could be calculated based on the emission quenching of the carbon dots. Likewise, a reversible moisture sensing paper was demonstrated, which changed its emission color from blue to yellow once placed in contact with an amount of 1 μL of water.

#### Bioimaging and other bio-applications

Circularly polarized light can be applied for bio-applications such as the detection of cancer cells^184^. This, together with an expanding interest in materials for microscopy and bioimaging with nonlinear properties, such as SHG^185,186^, chiral carbon dots have appeared at the forefront of several recent developments for bioimaging applications.

**Fig. 8:**
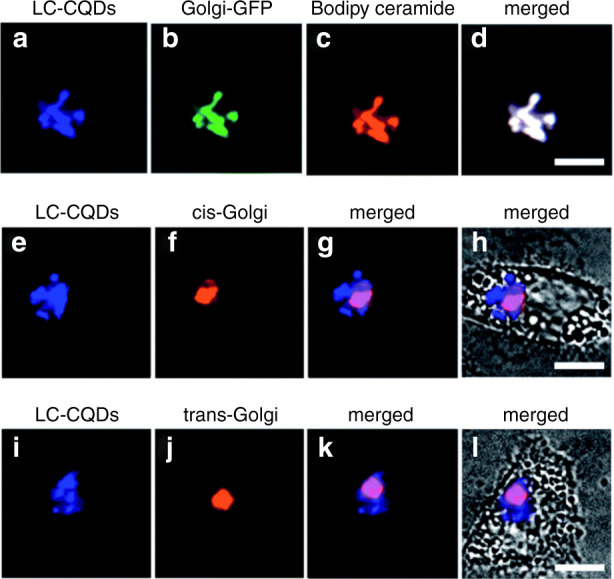
Targeting the Golgi apparatus with L-cysteine capped carbon dots. **a–c** Fluorescence image of the L-cysteine capped carbon dots (blue), Golgi-GFP (green, Golgi apparatus specific green fluorescent protein), and Bodipy ceramide (red, Golgi specific dye) in a human epithelial cell. **d** Merged image of **a–c**. **e** Fluorescence image of L-cysteine capped carbon dots. **f** Immunofluorescence image of the cis-Golgi. **g, h** Merged images of the fluorescence and bright field images. **i** Fluorescence image of L-cysteine capped carbon dots. **j** Immunofluorescence image of the trans-Golgi. **k, l** Merged images of the fluorescence and bright field images. (Scale 10 μm). Figure taken with permission from^142^ Copyright 2017 Royal Society of Chemistry

Li et al. showed that L-cysteine preferentially targets the Golgi apparatus in human epithelial cells (Fig. 8)^142^. Human cells were stained with L-cysteine capped carbon dots, whose emission was easily detectable under fluorescence microscopy. A high Pearson’s correlation factor of over 0.9 was reported for L-cysteine capped carbon dots, indicating their specific accumulation at the Golgi apparatus, with D-cysteine capped chiral carbon dots showing a much lower correlation factor of 0.35, highlighting the role of chirality. The importance of L-cysteine’s binding site specificity was further confirmed through experiments using L-cysteine attached to silica nanoparticles. The L-cysteine chiral carbon dots showed high photostability, sustaining continuous laser irradiation for one hour during which their PL signal dropped only by 10%. After infection of the cell with a respiratory syncytial virus, the fragmentation of the Golgi apparatus was visualized through fluorescence microscopy^142^. A similar study of the Golgi apparatus was undertaken with chiral carbon dots synthesized from L-/D-penicillamine and citric acid^187^.

Victoria et al.^57^ demonstrated potential antimicrobial effects of L-cysteine or D-cysteine capped chiral carbon dots. The addition of carbon dots reduced microbial levels and inhibited their growth at certain concentrations. Interestingly, different bacteria had different minimum concentrations for inhibition for L- or D-cysteine synthesized carbon dots. For example, L-cysteine carbon dots were more effective to inhibit *K. aerogenes ATCC 13048* strains, while D-cysteine carbon dots were more effective to inhibit *M. luteus DSM20030*, *B. subtilis DSM10, E. coli ATCC 25922*, and *E. coli MG1655* strains. This indicates that the stereochemistry of carbon dots plays an important role in microbial inhibition.

**Fig. 9:**
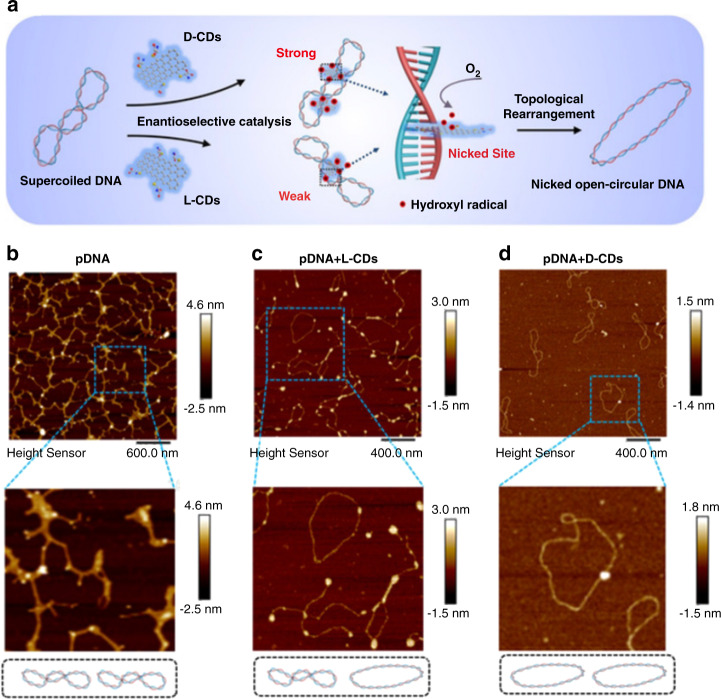
Enantioselective chiral carbon-dot-mediated topological rearrangement of supercoiled DNA. **a** Schematic illustration, and **b–d** AFM images of pristine pDNA (**b**), pDNA treated with l-cysteine capped CDs (**c**), and pDNA treated with d-cysteine capped CDs (**d**). Figure taken with permission from^61^ Copyright 2020 Wiley-VCH GmbH

Li et al. reported chiral carbon dots mimicking topoisomerase I enzymes (Fig. 9a–d)^61^. Topoisomerase plays an important role in the transcription of DNA to RNA, as it is able to cleave a strand of the DNA double helix and rearrange its nicked sites after relaxation^188^. As a sample DNA, plasmid PRSET-eGFP was used, with a length of 3579 bp. After incubation with L-cysteine-based carbon dots, the topological rearrangement was measured to be 55% after 24 h (Fig. 9c), while it was 91% when using D-cysteine-based carbon dots (Fig. 9d).

#### Catalysis, inhibition, and chiral induction

Zhang et al. highlighted a possible role of chiral carbon dots to enhance the growth of plants^139^. Mung beans were cultivated with solutions of different concentrations of L-cysteine or D-cysteine passivated carbon dots, as well as achiral ones. Those cultivated with D-cysteine capped carbon dots at a concentration of 100 μg/mL showed an increased carbohydrate content, indicating an increased ability to absorb water. Also, Ribisco enzyme activities seemed to be slightly increased, indicating a beneficial effect on mung bean growth^139^.

A similar study on cellular metabolism was undertaken by Li et al.^140^, who incubated T24 cells with L- and D-cysteine capped chiral carbon dots. In confocal microscopy it could be seen that the carbon dots were mostly absorbed by mitochondria and lysosomes. After treating cells with carbon dots for 24 h, a significantly increased glycolysis could be measured via the extracellular acidification rate, with the L-cysteine-based carbon dots inducing a much higher increase in glycolysis as compared to D-cysteine-based enantiomers.

**Fig. 10:**
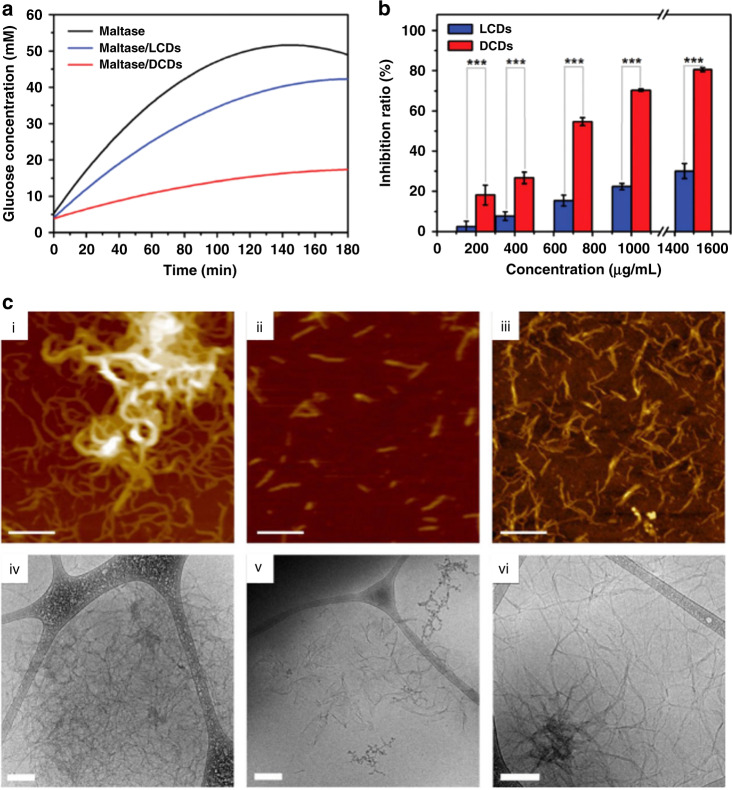
Examples of catalysis and inhibition with chiral carbon dots. **a** Glucose concentration resulting from the maltose degradation for maltase (black line), maltase/L-glutamic acid capped CDs (LCDs, blue line), and maltase/D-glutamic acid capped CDs (DCDs, red line) at different hydrolysis times, and **b** inhibition ratio of LCDs (blue trace) and DCDs (red trace) with different concentrations after 90 min reaction. **c** Inhibition of prion peptide fibril assembly with L-/D-lysine derived chiral carbon dots: (i–iii) AFM and (iv–vi) cryo-TEM images of the assembly on silicon wafer in the absence of carbon dots (i, iv), in presence of L-lysine-based carbon dots (ii, v), and in presence of D-lysine-based carbon dots (iii, vi). Bars correspond to 200 nm. **a, b** Adapted with permission from^68^ Copyright 2019 Wiley-VCH GmbH. **c** Adapted with permission from^145^ Copyright 2018 Wiley-VCH GmbH

Zhang et al.^68^ treated maltase with chiral carbon dots based on L- and D-glutamic acid and detected a reduction of up to 80% in glucose production in the case of D-glutamic-acid-based carbon dots, while L-glutamic-acid-based carbon dots only reached about 30% inhibition at the same concentration (Fig. 10a,b). The influence of carbon dots based on L- and D-cysteine on laccase activity was studied by Hu et al.^135^. In a water bath, the laccase activity was estimated from the degree of oxidation on a 2,2-azinobis (3-ethylbenzothiazoline-6-sulfonic acid) diammonium salt (ABTS) substrate, which led to an increased absorbance. The addition of L-cysteine capped CDs increased the absorbance by 20%, while the same amount of D-cysteine capped CDs decreased it by 10%. Chiral carbon dots as an enantioselective catalyst for glucose oxidase have been suggested as well^60^, with D-tyrosine-based carbon dots increasing glucose oxidase activity by 35%.

Amyloid diseases caused by prions can cause fatal medical conditions such as the Creutzfeldt–Jakob disease and Gerstmann–Straussler–Scheinker disease^189,190^ These diseases are thought to be caused by the conversion of prion peptides to the form PrP^Sc^ (scrapie isoform of the prion protein) which can form plaque in extracellular space around body tissues^189^. Arad et al. exploited L-/D-lysine-derived chiral carbon dots as inhibitors of prion peptide assemblies^145^. Amyloid aggregation was followed by detecting fluorescence of thioflavin-T, which increased in the presence of amyloids. The addition of chiral carbon dots significantly reduced this fluorescence, indicating inhibition of β-sheet formation, with L-lysine-derived carbon dots inducing a stronger quenching effect than D-lysine ones. AFM images showed much shorter and less entangled fibrils in the presence of L-lysine derived carbon dots (Fig. 10c(i–iii)), supported by TEM images shown in Fig. 10c(iv–vi). The authors attributed the inhibition of fibril formation to the chiral nature of carbon-dot/prion protein interaction, which interfered with prion protein–lipid interactions. The same group also studied the effect of such chiral carbon dots on the aggregation of amyloid β-protein Aβ-42^144^, which is a primary factor associated with Alzheimer’s disease^191^. The formation of peptide β-sheets was evidenced by the appearance of CD maxima and minima at 195 nm and 218 nm. While the presence of D-lysine-based carbon dots did not significantly change the CD spectra, L-lysine-derived carbon dots added to a solution of Aβ-42 prevented the formation of these characteristic CD peaks. Cryogenic TEM showed that L-lysine-based carbon dots attached to fibrils and prevented them from forming a fibrillar network. Cytotoxicity studies showed that Aβ-42 has reduced the cell viability by about 25% after 24 h, while the addition of L-lysine-based carbon dots prevented this effect.

Similar to aldol reaction catalysis with an aid of chiral ZnS quantum dots^173^, aldol reaction catalysis with chiral carbon dots have also been studied^152^. It was found that achiral carbon dots, as well as dialyzed D-proline molecules, did not yield any reaction product in a direct aldol reaction of cyclohexanone and o-nitrobenzaldehyde. On the other hand, chiral carbon dots synthesized from D-proline as precursor catalyzed this reaction with a 98% product yield, with the products having 73% enantiomeric excess, indicating their chiral catalytic activity^152^. However, the authors also mentioned that non-dialyzed D-proline molecules reached a product yield of 96% and an enantiomeric excess of 93%, indicating that the chiral carbon dots were only slightly better catalysts and their reaction products were in fact less chiral than those obtained using D-proline as a catalyst.

Chiral carbon dots can transfer their chirality to achiral molecules, as was shown by Liu et al.^141^. After incubating L- and D-cysteine-based chiral carbon dots with H_2_TPPS porphyrins, circular dichroism peaks at 420, 460, and 500 nm appeared, which were neither present in chiral carbon dots nor in H_2_TPPS.

To summarize our previous discussion, an overview of synthetic methods, morphology, chiroptical properties, and applications of chiral carbon dots is presented in Table 1.

**Table 1:** Overview of synthetic methods, morphology, chiroptical properties, and applications of chiral carbon dots

Chiral precursor	Other precursors	Synthetic method	Solvent	EM nm	Size nm	CPL/TPA	Application	Ref.
L-ascorbic acid	Copper (II) acetate	Heating	Water				Enantioselective sensing of ascorbic acid	^195^
L-aspartic acid	Citric acid	Hydrothermal	Water + NaOH	420	8		Enantioselective sensing of Sn^2+^ ions and L-lysine	^149^
Cellulose nanocrystals	Ethylene diamine	Heating	Water + boric acid	460	2–3	CPL		^63^
Cellulose nanocrystals	Carbon dots	NHS/EDC coupling	Water	420 550 650	6–12	CPL		^64^
L-/D-cysteine		Hydrothermal	Water + NaOH	460	4–5		Enantioselective sensing of L-/D-tartaric acid	^69^
L-/D-cysteine		Hydrothermal	Water + NaOH	460	4–5		Chiral self-assembly of porphyrins	^141^
L-/D-cysteine		Heating	Water	500			Enantioselective topological rearrangement of DNA	^61^
L-/D-cysteine		Heating	Water + NaOH	510	5–7		Enantioselective cell glycolysis catalysis	^140^
L-/D-cysteine		Electrochemical	Water + NaOH	485	4–5		Enantioselective laccase catalysis	^135^
L-/D-cysteine	Graphene quantum dots	NHS/EDC coupling	Water	520–550	2–7	CPL = 0		^53^
L-/D-cysteine	Citric acid	Hydrothermal	Water	418	2–6	TPA	Ink	^55,138^
L-/D-cysteine	Citric acid	Hydrothermal	Water	440	4–8		Mung bean growth catalysis	^139^
L-cysteine	Citric acid	Heating	Water	420	9		Imaging of Golgi apparatus	^142^
L-/D-cysteine	Citric acid	Microwave	Water	420	12		Enantioselective inhibition of bacterial growth	^57^
L-/D-cysteine	Citric acid + urea	Hydrothermal	Water	450	5	TPA		^62^
L-cysteine	Citric acid + EDA	Heating	Water	445	10		Enantioselective sensing of L-lysine	^182^
L-cysteine	p-Benzoquinone	Microwave	Ethanol	550	3		Moisture sensing	^148^
L-/D-cysteine	Copper (II) acetate	Heating	Water	450–520	6			^95^
L-/D-glutamine	Citric acid	Hydrothermal	Water	450	3–4		Sensing of Fe^3+^ ions	^183^
L-glutathione	Ethylene diamine	Hydrothermal	Water	390	2.5		Sensing of arginine	^143^
L-/D-glutamic acid		Electrochemical	Water + NaOH	405	3–7		Enantioselective inhibition of maltase activity	^68^
L-/D-glutamic acid L-methionine		Microwave	Water + NaOH	445				^151^
L-/D-glucose	Monosodium phosphate	Microwave	Water	450	3–7		Enantioselective sensing of L-/D-pencillamine	^71^
Guanosine 5’-monophosphate		Microwave	Water	430–460	5			^52^
L-/D-lysine	Jeffamine® ED-900	Hydrothermal	Ethylene glycol	450	2–3		Enantioselective inhibition of Aβ42 assembly	^144^
L-/D-lysine	Jeffamine® ED-900	Heating	Ethylene glycol	450	4		Enantioselective inhibition of prion peptide fibril assembly	^145^
L-/D-methionine D-glucose D-glucosamine L-aspartic acid L-alanine		Heating	No solvent	400–450	2–5		Interaction with Azobenzene	^150^
(R)/(S)-2-phenyl-1-propanol	Graphene quantum dots	Heating	No solvent	520	22			^54^
D-proline	Citric acid	Hydrothermal	Water	420	6		Enantioselective direct aldol reaction catalysis	^152^
(+)/(−)-sparteine	Sucrose	Microwave	Water	450–600	14		Enantioselective sensing of L-/D-cysteine and L-/D-proline	^146^
(R,R)/(S,S)-1,2-cyclohexanediamine	Arginine	Microwave	Water	425	3	CPL=0		^58^
D/L-tartaric acid + L/D-penicillamine		Heating	No solvent	441	2–5			^153^
L-/D-tartaric acid	Graphene quantum dots	Electrodeposition	Water		2.5		Enantioselective sensing of L-/D-tryptophan	^70^
L-/D-tryptophan		Hydrothermal	Water + NaOH	476	4			^56^
L-/D-tryptophan	o-Phenylenediamine	Solvothermal	Ethanol + HCL Water Water + H_2_SO_4_	450 560 610	2.5 3.0 4.5	CPL		^59^
L-/D-tyrosine	Citric acid	Heating	Water	450	3–7		Enantioselective glucose oxidase catalysis	^60^
L-/D-pencillamine	Citric acid	Heating	Water	440	1–4		Imaging of Golgi apparatus	^187^
L-/D-phenylalanine L-/D-cysteine L-/D-histidine L-/D-proline L-/D-tryptophan L-/D-alanine L-/D-proline methyl ester	Sucrose	Microwave + NHS/EDC coupling	Water	480	8–14			^147^

*EM* emission peak, *CPL* circular polarized light, *TPA* two-photon absorption

## Summary and outlook

Carbon dots have already proven to be an intriguing class of nanoparticles, which can be produced through simple fabrication methods, are low toxic or even biocompatible, and offer useful optical properties such as strong emission tunable over the visible to near-infrared spectral range^192^. Chiroptical properties together with the potential of multiple photon absorption and emission make these nanoparticles highly interesting in sensorics, bioimaging, and theranostics. In most cases, experimental approaches for the formation of chiral objects developed for colloidal nanoparticles, including semiconductor quantum dots, can be tailored for producing chiral carbon dots and nanostructures based on them. The literature analysis shows that chirality in carbon dots can be induced via several ways: (i) inheritance from chiral precursors with circular dichroism signals almost coinciding with that for pure precursors; (ii) intrinsic circular dichroism signals from the chiral carbon dots’ core; (iii) circular dichroism induced by chiral ligands or a chiral environment, and (iv) through the chiral assembly.

Despite the achievements that have been attained so far, research on chiral carbon dots is still in its infancy. While the influence of different synthetic parameters, including a variation of ligands and solvents, temperature, and reaction time on chiroptical properties of carbon dots have already been tested, more research is needed to clarify the underlying physical and chemical mechanisms of these phenomena. Thus, this research area still faces several challenges. Development of synthetic techniques including one-pot synthesis and post-synthetic functionalization of the surface is needed which would result in expanding the toolkit for controlling the chiroptical properties of carbon dots. Table 1 shows that many of the reported chiral carbon dots are in fact synthesized by incorporating a chiral ligand from a small pool of useable amino acids and other chiral molecules. Nonetheless, chiroptical data varies greatly. This shows an urgent need for the development of multiple synthetic pathways and a better understanding of formation mechanisms. Also, methods used to obtain other chiral nanostructures such as irradiation with circularly polarized light^193^ have not yet been reported for chiral carbon dots. Further advancement of chiral dot synthesis to achieve high absorption cross-sections for nonlinear processes such as two-photon absorption as well as circularly polarized emission is needed. Literature reports on these useful properties are still very sparse, while they are required to exploit applications in the biological window^194^. Since carbon dots endowed with chiral properties have already been shown to be useful in bio and medical applications, including advanced treatments of neurodegenerative diseases such as Alzheimer’s, the Creutzfeldt–Jakob disease, and Gerstmann–Straussler–Scheinker disease^144,145^, they could lead to new medical treatments. However, most of these studies are still in their infancy, and require a further extension in order to enter the real-world application and use in theranostics.
